# Aldehydes in Exhaled Breath during E-Cigarette Vaping: Pilot Study Results

**DOI:** 10.3390/toxics6030046

**Published:** 2018-08-07

**Authors:** Vera Samburova, Chiranjivi Bhattarai, Matthew Strickland, Lyndsey Darrow, Jeff Angermann, Yeongkwon Son, Andrey Khlystov

**Affiliations:** 1 Organic Analytical Laboratory, Desert Research Institute (DRI), Reno, NV 89512, USA; Chiranjivi.Bhattarai@dri.edu (C.B.); Yeongkwon.Son@dri.edu (Y.S.); Andrey.Khlystov@dri.edu (A.K.); 2 School of Community Health Sciences, University of Nevada, Reno, NV 89557, USA; mstrickland@unr.edu (M.S.); ldarrow@unr.edu (L.D.); jeffa@unr.edu (J.A.)

**Keywords:** aldehydes, breath analysis, e-cigarette emissions, respiratory tract retention, exposure

## Abstract

Several studies have shown the presence of aldehydes (i.e., formaldehyde, acrolein) in mainstream emissions of some e-cigarettes. For this reason, concerns have been raised regarding potential toxicity. The purpose of this research was to measure levels of carbonyls in exhaled breath of e-cigarette users during “vaping” sessions and estimate the respiratory tract (RT) uptake of specific aldehydes, including formaldehyde and acetaldehyde. We measured concentrations of 12 carbonyls in e-cigarette aerosols produced directly by e-cigarettes and in the exhaled breath of 12 participants (19 sessions). Carbonyls were sampled on 2,4-dinitrophenylhydrazine (DNPH) cartridges and analyzed with high performance liquid chromatography (HPLC) coupled with a UV/Vis photodiode detector. We found that in most cases, levels of aldehydes and methyl ethyl ketone (MEK) were significantly higher (2–125 times) in exhaled e-cigarette breaths than in pre-exposed breath. Exposure levels for the most abundant individual carbonyls in e-cigarette emissions—formaldehyde, acetaldehyde, acrolein—were between the limit of quantification (LOQ) and 24.4 μg·puff^−1^. The mean retention of formaldehyde in the respiratory tract was 99.7 ± 0.9% for all participants, while acetaldehyde retention was 91.6 ± 9.9%. Within the limitation of a small number of participants, our results showed that there is an increase in breath carbonyls during e-cigarette use.

## 1. Introduction

An electronic cigarette (e-cigarette) is a nicotine delivery device that has become one of the most popular alternatives to conventional tobacco cigarettes in recent years [1,2,3]. This device produces aerosolized nicotine in vapor form (e-vapor) by heating e-cigarette liquid (or e-liquid), which is typically composed of propylene glycol (PG), vegetable glycerin (VG), nicotine, and flavoring compounds [4]. A number of studies have shown that in addition to nicotine and flavorings, e-cigarette vapors may also contain carbonyl compounds, including potentially harmful species such as formaldehyde, acetaldehyde, and acrolein [5,6,7,8,9] as well as diacetyl [10]. Although many studies have reported aldehyde emissions from e-cigarettes, there are ongoing debates within the scientific, tobacco control, and tobacco manufacturing communities about whether these compounds are present in sufficient quantities in inhaled vapor to be harmful to e-cigarette users. Variability in these quantities can be explained by the difference in tested e-cigarette devices (type of coil, power output, and composition of flavored liquid) that causes a large variability in concentrations of emitted carbonyls [5,11,12]. Some investigators have argued [13] that dangerously high aldehyde concentrations in mainstream e-cigarette aerosols occur only during so-called “dry puff” conditions that are avoided by users because of the associated acrid taste, thus eliminating or minimizing aldehyde exposure during realistic e-cigarette use. However, high concentrations of aldehydes have been detected in e-cigarette emissions that have no option of power control (e.g., CE4 or V2) [11] and at power settings typically selected by e-cigarette users. Therefore, it is critical to further evaluate aldehyde e-cigarette exposure to better understand toxicological significance.

To our knowledge, research on human respiratory track (RT) retention of carbonyls, specifically formaldehyde and acetaldehyde, during e-cigarette use is lacking. RT uptake of aldehydes has been studied for conventional cigarettes [14,15], but the retention of aldehydes in e-cigarette users’ RT could differ from that of cigarette smokers. Large amounts of PG/VG aerosols can cause certain aldehyde compounds to partition into the particle phase, thus modifying RT retention efficiency. Long et al. [16] performed analysis of carbonyls in exhaled e-vapors and found no significant difference between exhaled e-cigarette breath. However, considering that mainstream e-cigarette carbonyls were not measured in the Long study, the exposure could not be estimated, and the low levels of carbonyls in exhaled e-cigarette breaths are most likely because of high carbonyl retention rates (above 95%) in the human RT [17,18].

The goal of this study was to estimate the extent to which carbonyl exposures occurred during realistic e-cigarette use conditions. With the limited number of participants, we aimed to determine if levels of carbonyls, including potentially harmful compounds such as formaldehyde and acrolein, were elevated in exhaled breath of e-cigarette users and confirm that carbonyl’s formation is not a laboratory artifact. For this purpose, concentrations of 12 aldehydes and butanone (methyl ethyl ketone [MEK]) were measured in mainstream and exhaled e-cigarette aerosols under real-life conditions and then accessed for carbonyl retention in participants’ RT.

## 2. Experimental

### 2.1. Materials

Carbonyl standards were purchased from AccuStandard, Inc. (New Haven, CT, USA). Acetonitrile (high performance liquid chromatography grade) was obtained from Fisher Scientific (Fair Lawn, NJ, USA). High purity grade water (18 MΩ·cm^−1^) was produced using a NanoPure system (Barnstead, Thermo Scientific, Dubuque, IA, USA). Cartridges loaded with 2,4-dinitrophenylhydrazine (DNPH, Sep-Pak DNPH-Silica Short Body Cartridges, part WAT047205) were obtained from Waters Corporation (Milford, MA, USA). Aerosol breath bags were purchased from Allied Healthcare Products, Inc. (St. Louis, MO, USA). Air (ultra-zero grade) was provided by Airgas, Inc. (Radnor, PA, USA). Detailed descriptions of e-cigarette devices and used e-liquid are summarized in Table S1 (Supplementary Material).

### 2.2. Participants

Twelve e-cigarette users (seven females and five males) in the age range of 21 to 65 years were recruited for sampling background and exhaled e-cigarette aerosol breaths (Table 1). The protocol for the collection of human breath (study ID number: 994577-1) was approved by the University of Nevada, Reno (UNR, Reno, NV, USA) Office of Human Research Protection (OHRP, Reno, NV, USA), approval date: 14 June 2016. One male volunteer participated seven times and one female volunteer participated two times using different e-cigarette devices or e-liquids (Table 1). Therefore, we had 19 paired samples of background breath and exhaled e-cigarette breath. All participants were asked not to vape at least two hours prior to breath collection, and no other specific limitations were required. Participants used their own e-cigarette devices and e-liquids except sessions #6–10. Participants of sessions #6–10 used a brand new e-cigarette provided in the laboratory (Table 1). Each volunteer signed a written informed consent approved by the local UNR institutional review board (IRB, UNR, Reno, NV, USA).

### 2.3. Sampling and Measurements

Breath sampling was conducted using the sampling setup presented in Figure S1 (Supplementary Material). The participants were asked to exhale their breath into a disposable 700 mL aerosol breath bag (Blowout Medical LLC, Evanston, WY, USA) using an exchangeable sterile mouthpiece. A sterile, one-way valve was incorporated between mouthpiece and air bag connected to the rest of the sampling system, such that participants were not able to inhale back anything from the sampling system. The exhaled breath was immediately pumped from the bag to minimize loss of exhaled carbonyls. The sample was pulled though the DNPH-coated cartridge with a flow rate of ~1 L·min^−1^. All samples were collected under the same conditions (flow rate, sampling system, type of air bag, sampling media, etc.) in the same laboratory room to minimize variation in inhaled background air and errors between samples. Before the vaping session, background breath was collected for each participant. Five breaths were sampled into one DNPH cartridge, and 2–3 replicate cartridges were collected. Exhaled e-cigarette breaths were collected the same way.

We collected mainstream e-cigarette emissions using an approach similar to the exhaled breath collection sampling system (Figure S1b), and it is described in Khlystov and Samburova [11]. Briefly, the operator/participant manually depressed the e-cigarette power button while the laboratory operator simultaneously switched a stainless steel, three-way valve to sample position (Figure S1b). The sample air was drawn by a pump through a mass flow controller (MassTrak 810C-DR-13-V1S0, Sierra Instruments Inc., Monterey, CA, USA). The puff duration during the sampling of the direct e-cigarette emissions varied between subjects and it was 3 ± 1 s on average. All samples were collected in triplicates (3 DNPH cartridges) with 3 puffs per one DNPH cartridge. However, to accurately measure direct emissions from tested e-cigarettes and thus subjects’ exposure, it was important to know the vaping topography parameters such as flow rate, puff duration, and puff profile. To investigate how flow rate and puff duration affect aldehyde emissions, additional experiments were performed. We tested an e-cigarette (Aspire Cleito) at three flow rates (0.4, 1.0, and 1.5 L·min^–1^) and three typical puff durations: 2, 3, and 4 s [19,20]. We found that the amount of emitted aldehydes was insensitive to flow rate but increased linearly with puff duration (data not presented). Aldehyde amounts emitted during a 4-s puff were no more than three times higher than during 2-s puffs. Given the common puff duration range [19], this represented about 50% maximum uncertainty. To minimize this uncertainty, we asked participants to manually depress the e-cigarette power button. For all subjects, the puff duration was within 2 and 4 s. The samples were collected with a flow rate of 0.4 L·min^–1^.

Collected DNPH cartridges were kept at 4 °C immediately after sampling, eluted within two hours to avoid chemical transformations of unsaturated carbonyls [21], and analyzed within 24 h with high performance liquid chromatography (HPLC, Waters 2690 Alliance System, Milford, MA, USA) coupled with a UV/Vis detector (Waters 996 photodiode array detector). A detailed description of the analytical method is in Khlystov and Samburova’s work [11]. Briefly, collected cartridges were eluted with 2 mL of acetonitrile and analyzed for 12 aldehydes (formaldehyde, acetaldehyde, acrolein, propionaldehyde, crotonaldehyde, methacrolein, n-butyraldehyde, benzaldehyde, valeraldehyde, glyoxal, m-tolualdehyde, hexaldehyde) and one ketone (MEK) by HPLC-UV/Vis detector. The compounds were separated on a Polaris HPLC column (C18-A, 100 × 2.0 mm, particle size: 3 µm) and quantified based on six-point external calibration for each analyte with an *R*^2^ value above >0.99 (median value of error for each curve point was ~5% for all analyzed carbonyls). The limit of detection (LOD) values were in the range of 0.001–0.01 µg·puff^−1^ (or µg·breath^−1^).

## 3. Results

### 3.1. Mainstream Concentrations

Table 1 summarizes concentrations of carbonyl compounds detected in aerosols sampled directly from participants’ e-cigarettes. The content of carbonyls varied among e-cigarette devices and e-liquid flavors [11]. Formaldehyde and acetaldehyde were the most abundant carbonyls detected in all e-cigarette vapor samples, ranging from 0.059 ± 0.006 to 24.4 ± 2.3 µg·puff^−1^ and from 0.022 ± 0.008 to 22.5 ± 6.2 µg·puff^−1^, respectively. The highest concentrations of formaldehyde and acetaldehyde were generated by the CE4 e-cigarette with Bubble Gum flavored e-liquid. Acrolein, glyoxal, and propionaldehyde were above their LOD in more than one half of the collected samples, and their concentration levels were from 0.012 ± 0.003 to 1.37 ± 0.35 µg·puff^−1^, from 0.019 ± 0.004 to 1.62 ± 0.39 µg·puff^−1^, and from 0.019 ± 0.008 to 4.2 ± 1.2 µg·puff^−1^, respectively. Overall, the highest concentration of total aldehydes and MEK were observed for the CE4 e-cigarette (0.97–53.3 µg·puff^−1^), while BLU and V2 e-cigarettes generated lower aldehyde levels (0.4–14.1 µg·puff^−1^), in good agreement with results from other studies [8,22].

We detected benzaldehyde in seven out of 16 e-cigarette vapor samples in the range of 0.11 ± 0.03 and 3.9 ± 1.2 µg·puff^−1^. Concentrations of eight carbonyls (crotonaldehyde, methacrolein, butyraldehyde, methylglyoxal, valeraldehyde, m-tolualdehyde, and hexaldehyde) were below their LODs. All of the detected aldehydes have been previously found in e-cigarette mainstream samples [5,11,23,24]. Although concentrations of individual compounds varied from device to device, our results are consistent with previously reported data [8,12]. For example, concentrations of formaldehyde, acetaldehyde, and acrolein measured in our study (Table 1) were within the range presented in Gillman et al. [5], where five different e-cigarette devices were tested at various power levels and 0.07–51 µg·puff^−1^ of formaldehyde, 0.03–41 µg·puff^−1^ of acetaldehyde, and 0.02–5.5 µg·puff^−1^ of acrolein were detected in direct e-cigarette emissions.

### 3.2. Concentrations in Exhaled E-Cigarette Breath

Concentrations of 12 aldehydes and MEK were measured in participants’ breath prior to each session (background breath or *C_background_*) and in exhaled e-cigarette breath (*C_e-cig breath_*). Background formaldehyde concentrations ranged between being below LOD and 0.012 ± 0.003 µg·breath^−1^ (mean: 0.003 ± 0.004 µg·breath^−1^). Background levels of acetaldehyde were higher than formaldehyde levels, in the range of 0.002–0.035 µg·breath^−1^ (mean: 0.015 ± 0.009 µg breath^−1^). The measured background levels of carbonyls were compared with those in exhaled e-cigarette breaths.

**Figure 1:**
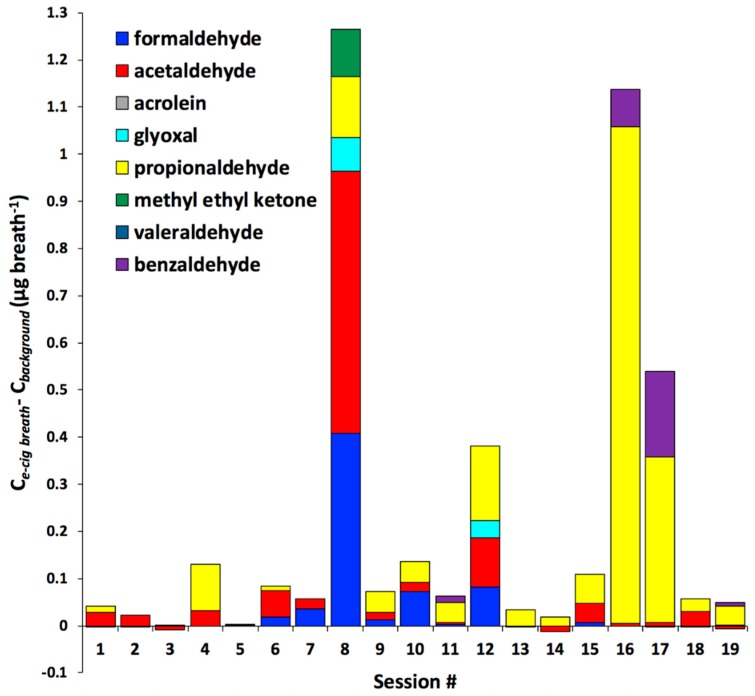
Difference in carbonyl concentrations between exhaled e-cigarette breath (*C_e-cig breath_*) and background breath (*C_background_*); units: µg breath^−1^; the concentrations are also presented in Table S2.

Figure 1 (Table S2) shows differences between carbonyl concentrations in exhaled e-cigarette breath relative to background levels (∆*C = C_e-cig breath_ − C_background_*; units: µg breath^−1^). In 14 out of 19 sessions, total concentrations of aldehydes and MEK were higher in exhaled e-cigarette breath (∆*C* > 0) than those in the background breath. We detected a factor of 1.4 to 53 increase (factor of 13 on average) above the formaldehyde background level in aerosols exhaled in seven sessions (#6–10, 12, and 15), where the highest ∆*C_formaldehyde_* values were observed for participants in sessions #8 (0.4 µg·breath^−1^), #10 (0.07 µg·breath^−1^), and #12 (0.08 µg·breath^−1^). Note that formaldehyde concentration levels were found to be hundreds of times higher in direct e-cigarette emissions (Table 1) than in exhaled e-cigarette breaths (Figure 1). This large difference between mainstream aerosol and breath formaldehyde levels is most likely because of the high retention of the formaldehyde in the users’ RT [14,17]. Deviations in vaping topography during e-cigarette use by volunteers and during collection of vapors directly from e-cigarettes can also contribute to the observed differences in aldehyde concentrations between exhaled and mainstream aerosols. As discussed in “*Sampling and Measurements*”, however, errors in reproducing topography are not more than a factor of two, especially given that during mainstream aerosol measurements, the participants were asked to reproduce puff durations that they normally use during vaping. We calculated the RT aldehyde uptake for the two most abundant aldehydes (acetaldehyde and formaldehyde) in e-cigarette emissions [6,8] and present these results in Figure 1.

Concentrations of acetaldehyde for the majority of participants were higher in exhaled e-cigarette breaths (1.2–62 times; mean: 8.9) than in background breaths with ∆*C_acetaldehyde_* from 0.003 ± 0.015 to 0.56 ± 0.11 µg·breath^−1^. The highest acetaldehyde concentration in exhaled e-cigarette breath was observed for participants in sessions #8 and 12, where ∆*C_acetaldehyde_* values were 0.56 ± 0.11 and 0.10 ± 0.02 µg·breath^−1^, respectively (Figure 1, Table S2). Similar to formaldehyde, acetaldehyde concentrations in mainstream e-cigarette vapors were higher (~50 times on average), which is most likely because of great absorption of acetaldehyde in participants’ RT [14,17,18].

We also observed higher concentrations in exhaled e-cigarette breath samples than in background breath samples for propionaldehyde (Figure 1). In 15 of the 19 sessions, ∆*C_propionaldehyde_* was positive and ranged from 0.010 ± 0.002 to 1.05 ± 0.08 µg·breath^−1^. For sessions #2, 3, 5, and 7, no propionaldehyde was detected in either background or exhaled e-cigarette breath samples. Propionaldehyde is one of the possible products of thermal decomposition of flavoring compounds that was detected in vapors emitted by e-cigarettes [8,11]. Breaths of participants during sessions #12 (∆*C_propionaldehyde_* = 0.16 µg·breath^−1^), #16 (∆*C_propionaldehyde_* = 1.05 µg·breath^−1^), and #17 (∆*C_propionaldehyde_* = 0.35 µg·breath^−1^) contained greater levels of propionaldehyde relative to other sessions (Figure 1, Table S2). At the same time, high propionaldehyde concentrations were measured in direct emissions of e-cigarette devices used by volunteers in sessions #12 (0.19 ± 0.04 µg·puff^−1^), #16 (12.1 ± 2.7 µg·puff^−1^), and #17 (0.18 ± 0.04 µg·puff^−1^) (Figure S2, Supplementary material). In comparison, the propionaldehyde level in direct e-cigarette emissions for the rest of cases (except e-cigarette #8) was lower, in the range of 0 to 0.10 ± 0.02 µg·puff^−1^. Although it seems like there is an association between high propionaldehyde concentration in direct e-cigarette emission and elevated propionaldehyde level (∆*C_propionaldehyde_*) in participants’ e-cigarette exhaled breath, no significant correlation was observed (Spearman *r* = 0.16, *p* = 0.53).

We detected several aldehydes (benzaldehyde and glyoxal) only in exhaled e-cigarette breaths, while being below LOD in all background breath samples. Benzaldehyde is one of the flavoring compounds that is widely used in e-cigarette liquids [4,7]. It was detected in exhaled e-cigarette breaths (∆*C_benzaldehyde_*) of #11, 16, 17, and 19 samples ranging between 0.007 and 0.18 µg·puff^−1^. Glyoxal, an aldehyde with acute toxic effects [25], has been detected in the mainstream of many e-cigarette devices [11,23], including e-cigarettes tested in this study (Table 1). Glyoxal was found in exhaled e-cigarette breaths of two subjects (sessions #8 and 12) and was below LOD in background breath. Interestingly, in mainstream e-cigarette emissions, glyoxal was below LOD in only five out of 19 (Table 1) samples, meaning that absorption of this aldehyde by RT is close to 100% in the majority of cases. Acrolein is another potentially hazardous carbonyl compound, the inhalation of which can cause severe pulmonary diseases [26,27]. We detected acrolein in 12 mainstream e-cigarette samples (Table 1), but its concentration was below LOD for all breath samples pointing to high absorption of acrolein by human RT.

Overall, the variation of aldehydes and MEK levels in participants’ breath varied substantially (Figure 1). This variability can be explained by the following factors: (i) use of different e-liquid flavors and e-cigarette devices; (ii) variability in age, gender, physical condition, and lung function of participants; (iii) difference in participants’ vaping style.

### 3.3. Exposure and RT Retention

**Figure 2:**
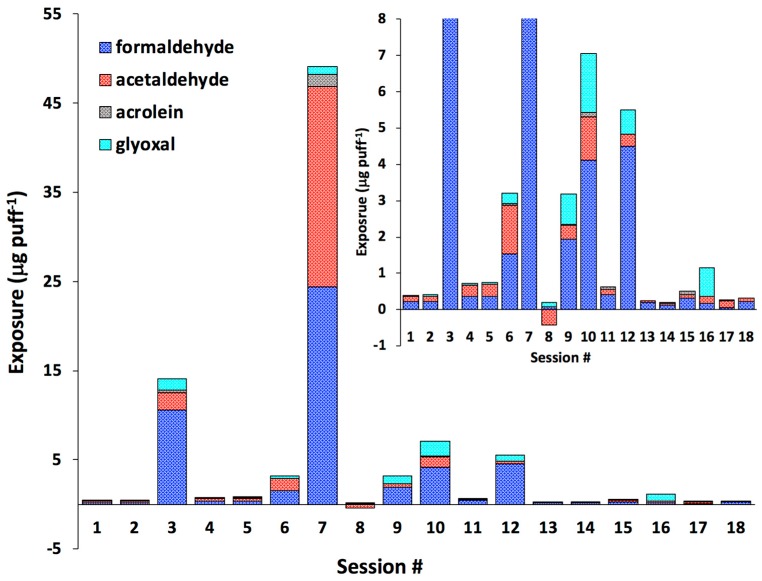
Level of exposure to selected carbonyls (formaldehyde, acetaldehyde, acrolein, and glyoxal); data are not available for session #19; these results are also presented in Table S3.

Next we examined the level of exposure by calculating the difference between aldehyde concentrations in mainstream e-cigarette emissions (*C_mainstream_*, µg puff^−1^) and elevated aldehyde levels in exhaled breath (∆*C*) during e-cigarette use (Figure 2, Table S3). The highest levels of exposure to total aldehydes and MEK were observed for sessions #3 (14.2 µg·puff^−1^), #7 (53.2 µg·puff^−^^1^), and #16 (12.8 µg·puff^−1^). For formaldehyde and acetaldehyde, we found the highest exposure levels in six cases (sessions #3, 6, 7, 9, 10, and 12) in the concentration range of 0.33–24.4 µg·puff^−1^. Exposure to acrolein was observed in 12 out of 18 sessions, ranging between 0.01 and 1.4 µg·puff^−1^ (Figure 2, Table S3).

In order to estimate how much aldehyde was retained in human RT, we calculated the aldehyde retention fraction (*F_RT_*) using the following formula:

$$F_{RT}=\frac{C_{mainstream}-\Delta C}{C_{mainstream}}$$

where *F_RT_*—fraction of aldehyde retained by RT, *C_mainstream_*—aldehyde concentration measured in direct e-cigarette emissions, ∆*C*—concentration of aldehyde in subject’s breath during vaping (∆*C = C_e-cig breath_ − C_background_*).

**Figure 3:**
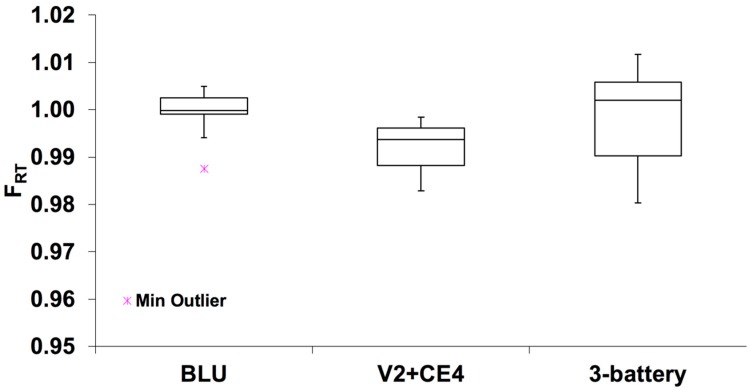
Formaldehyde retained by participants’ respiratory tracts (RTs). Error bars represent minimum and maximum values; boxes represent upper (75%) and lower (25%) quartiles, midline—median value.

Figure 3 shows the retention fraction of inhaled formaldehyde for three groups of participants: (a) users of BLU personal e-cigarette devices, (b) participants who were asked to use unfamiliar e-cigarette devices (V2 or CE4), and (c) participants who used their personal three-battery vaping devices (Aspire Cleito and Sigelei). As can be seen for all three groups, the amount of formaldehyde retained by RT was above 97% with mean percentile values of 99.8 ± 0.6% (BLU), 99.2 ± 0.8% (V2 + CE4), and 99.8 ± 1.2% (Aspire Cleito and Sigelei). Such a significant uptake of formaldehyde was expected since it is a highly water-soluble compound and thus is well retained by the RT hydrophilic surface. Our results are in good agreement with previously reported data. For example, Overton et al. [18] used two dosimetry models and predicted that more than 95% of the inhaled formaldehyde would be retained in the RT. Close to 100% of formaldehyde uptake was also reported by J.L. Jr. Eagle [28], who measured formaldehyde in dog RTs. Moldoveanu et al. reported 95–100% formaldehyde RT retention values for cigarette smokers [15].

**Figure 4:**
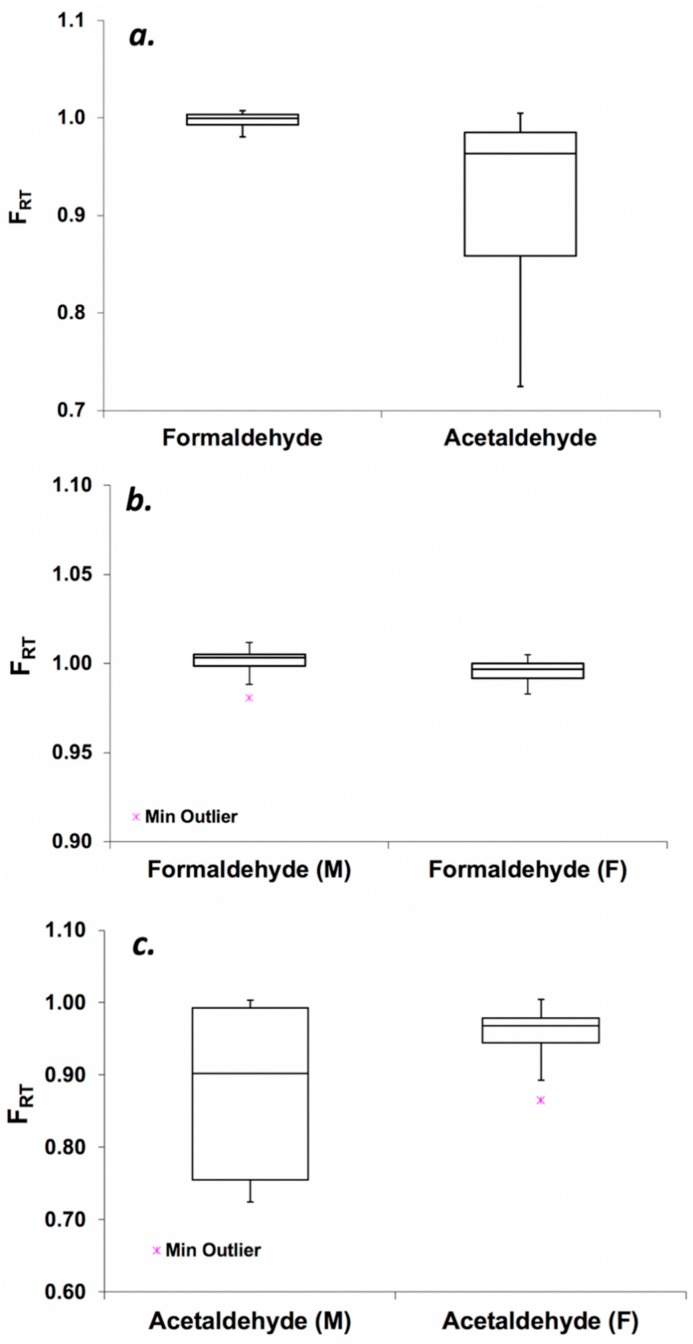
Fraction (*F_RT_*) of aldehydes retained by human RTs: (**a**) *F_RT_* of acetaldehyde and formaldehyde measured for all participants; *F_RT_* of (**b**) formaldehyde and (**c**) acetaldehyde for male (M) and female participants (F).

Although no significant difference in formaldehyde uptake among three groups of participants (Figure 3, *p* > 0.21) was observed in our study, slightly lower formaldehyde retention was observed in the second group (V2 and CE4 users). Participants in this group were asked to vape an e-cigarette and e-liquid that was unfamiliar to them. Although we do not have puff topography measurements, we observed that group 2 participants were cautious to deeply inhale the unfamiliar flavor generated by a new e-cigarette device. We suspect that an unfamiliar e-cigarette was the reason for the slightly lower formaldehyde uptake. Overall, the mean value of formaldehyde RT retention for all participants was 99.7 ± 0.9% (Figure 4a). In the case of acetaldehyde, average uptake by the RTs was 91.6 ± 10% with minimum and maximum values 72.4 and 100%, respectively (Figure 4a). Except for session #7 (uptake: 72.4%), retention of acetaldehyde in the RT was found to be above 75% for all participants’ sessions. No significant difference in formaldehyde (*p* = 0.36) and acetaldehyde (*p* = 0.09) RT retention was observed between female and male participants (Figure 4b,c).

Compared to formaldehyde, acetaldehyde RT uptake was lower, which can be explained by acetaldehyde’s lower water solubility (~400-fold lower than formaldehyde). Moreover, the presence of formaldehyde in particulate phase (mainly in PG/VG aerosols) [29] may increase RT retention of this aldehyde. To our knowledge, there is limited research on pulmonary retention of acetaldehyde in either humans or animals. In 1969, Dalhamn et al. [14] presented retention of different compounds, including acetaldehyde, in RTs during cigarette smoking and showed a 99 ± 1.2% acetaldehyde RT uptake. This value is about 7.5% higher than medium acetaldehyde uptake measured in our study. The RT retention of acetaldehyde reported by Moldoveanu et al. [15] for conventional cigarettes (94–99%) is close to our values but still above average RT uptake (91.6 ± 10%). The difference in acetaldehyde RT retention during e-cigarette use can be explained by the presence of PG/VG particles in e-cigarette aerosol that could affect gas-particle phase partitioning of acetaldehyde and, therefore, its deposition mechanism in the human pulmonary system. Moreover, smoking and vaping topographies are different [30], which could also affect RT uptake of aldehydes. For example, several studies [31,32] showed that puff durations for e-cigarettes are longer than those for conventional cigarettes. In addition, a different vacuum is needed for e-cigarette activation than for smoking traditional cigarettes [33]. Thus, intake of e-cigarette aldehydes and associated health effects cannot be extrapolated using data on conventional cigarettes, and assessment of “real-world” e-cigarette exposure is important. 

### 3.4. Mainstream Aldehydes vs. ∆C_aldehyde_

We performed a comparison between elevated aldehyde concentrations in exhaled breath during e-cigarette use (∆*C_aldehyde_*) and mainstream e-cigarette aerosols for the three most abundant aldehydes in all samples: formaldehyde, acetaldehyde, and propionaldehyde. A positive correlation was observed for formaldehyde with Spearman *r* of 0.76 (*p* = 0.0003). Unlike formaldehyde, we found no apparent correlation between elevated exhaled acetaldehyde (Spearman’s *r* = 0.10, *p* = 0.70) during vaping (∆*C_acetaldehyde_*) and direct acetaldehyde emissions from e-cigarettes. No significant correlation was observed for propionaldehyde (Spearman *r* = 0.16, *p* = 0.53) either. The poor correlation is perhaps because of the limited number of recruited participants and use of different e-cigarette devices and flavoring liquids. For this reason, we compared the same correlations (Table S5) within each group of e-cigarettes (Table 1): (i) BLU and V2 (sessions #1–6), (ii) CE4 (sessions #7–10), and (iii) three-battery vaporizers Aspire Cleito and Sigelei (sessions #11–19). For BLU and V2 e-cigarettes, a positive Spearman’s “Mainstream aldehydes vs. ∆*C_aldehyde_*” correlation was observed only for formaldehyde (*r* = 0.948, *p* = 0.013). A positive formaldehyde correlation was also found for the three-battery vaporizers Aspire Cleito and Sigelei (*r* = 0.695, *p* = 0.056). In the case of the CE4 device, no significant correlations were found for all three aldehydes (−0.800 < *r* < −0.02, *p* > 0.330).

## 4. Discussion

Our results showed that concentrations of analyzed carbonyls were higher in exhaled e-cigarette breaths than in background breaths in the majority of participants’ sessions. The total carbonyl concentration, on average, was 10.5 times higher in exhaled e-cigarette breaths than in background breaths. Our results clearly showed that high carbonyl concentrations—including those of potentially hazardous formaldehyde, acetaldehyde, and acrolein—were not limited to dry puff conditions [13], since participants were using their e-cigarettes in their typical “vaping” style. None of the participants using their own or the provided e-cigarette with a flavored e-liquid complained of unpleasant sensations during vaping sessions. The only complaint was received from a participant who was offered unflavored pure PG/VG liquids that were found to be “unpleasant.” High RT uptake of acetaldehyde (mean: 91.6 ± 9.9%) and formaldehyde (mean: 99.7 ± 0.9%) was obtained for all cases, and no significant difference was observed for RT uptake of these aldehydes between male and female participants. High exposure to formaldehyde (1.53–24.4 μg·puff^−1^; mean: 7.8 μg·puff^−1^) was observed in six (out of 18) cases, and the mean value of these exposure levels is comparable with exposure to conventional cigarette formaldehyde (~5 μg·puff^−1^) [34]. The Acute Exposure Guideline Levels (AEGL-1) for formaldehyde, acetaldehyde, and acrolein are 1.1, 81, and 0.070 mg·m^−3^, respectively, for 10 min exposure [35]. We converted our aldehyde levels into mg·m^−3^ for 10 min exposure (Supplementary Material, Table S4) and found that formaldehyde concentrations were above the AEGL-1 for sessions #3 (1.93 mg·m^−3^) and #7 (4.44 mg·m^−3^) and were close to the AEGL-1 for participants’ sessions #10 (0.76 mg·m^−3^) and #12 (0.84 mg·m^−3^). Acetaldehyde levels didn’t exceed the AEGL-1 for any participants. In the case of acrolein, the exposure level (0.250 mg·m^−3^) was 3.6 times higher than the AEGL-1 for participant session #7.

The observed large variability in aldehyde concentrations was most likely because of differences in e-cigarette conditions (type of e-liquid and e-cigarette, e-cigarette settings) and volunteers’ vaping styles (or vaping topography).

The present study has several limitations. First, the sample size was rather limited, considering the observed variability among participants in their vaping styles, used e-cigarettes, and e-liquid flavors. Twelve e-cigarette users were recruited; one male and one female participant were engaged seven and two times, respectively. Thus, 19 experimental sessions were performed during the study (Table 1). The sample size was sufficient, however, to detect a significant increase in aldehydes and MEK concentration in exhaled e-cigarette breaths relative to background breaths. Second, the puff duration of individual participants was measured with a timer as no topography devices were available, making puff duration measurements less accurate (±1 s). Among all participants, the puff duration varied from 2 to 4 s. Given a linear dependence of carbonyl emissions on puff duration and that the mean puff duration was 3 s, our estimates of inhaled carbonyls could be up to 50% uncertain. In order to reduce this uncertainty during the sampling of mainstream e-cigarette emissions, we asked participants to manually depress the e-cigarette power button for the duration they use when vaping. This way, the puff duration during e-cigarette use by a participant is expected to be close to the puff duration for direct e-cigarette emissions generation, thus significantly reducing the uncertainty. We need to emphasize that in future studies, it is important to use a vaping topography device to minimize the uncertainty in carbonyl generation during e-cigarette use. Third, no losses of breath aerosols onto sampling bag walls (Figure S1a) or chemical transformations undergone by carbonyls during the sampling were evaluated. To avoid the chemical transformation of unsaturated carbonyls [21], the samples were eluted within two hours after the sampling and analyzed within 24 h. Another limitation in relation to overall health impact assessment was that this study focused only on analysis of aldehydes, while other chemicals (e.g., toluene, lead, naphthalene, flavorings) have also been found in e-cigarette vapors [36,37] and may have a substantial impact on human health. In addition, our recent experiments with DNPH cartridges and DNPH impregnated filters showed that even though the DNPH-cartridge is an effective medium to collect gas-phase carbonyls [38], levels of particle phase carbonyls can be underestimated (~30%). More details on efficiency of different sampling media to collect gas and particle phase e-cigarette carbonyls will be presented in a following paper.

## 5. Conclusions

This pilot study underlines a potential health risk associated with carbonyls (i.e., formaldehyde, acetaldehyde, acrolein) generated by e-cigarettes. Concentrations of 12 aldehydes and MEK were measured directly in exhaled e-cigarette breaths of human volunteers, and RT uptakes were estimated for the most abundant in e-cigarette emissions carbonyls (formaldehyde and acetaldehyde).

Results of this study suggest: (1) concentrations of carbonyls, such as formaldehyde and acetaldehyde, are higher (2–125 times) in exhaled e-cigarette aerosols than in background breath of e-cigarette users, (2) since most of the recruited volunteers used their personal e-cigarette devices, this study confirms that significant amounts of carbonyls are indeed produced during normal e-cigarette use and that high carbonyl emissions observed in numerous laboratory studies [5,6,8,9] cannot be dismissed as laboratory artifacts, (3) e-cigarette aldehyde exposure needs to be assessed in future studies that include a larger set of participants and (4) for an accurate health risk assessment, it is important to correlate aldehyde exposure with the “vaping topography”, type of e-cigarette, e-cigarette settings, and chemical composition of e-liquids.

## Figures and Tables

**Table 1:** A summary of data on participants and e-cigarettes.

Session #	Participant #/Gender	E-Cigarette	Flavor	Formaldehyde	Acetaldehyde	Acrolein	Glyoxal	Propionaldehyde	Benzaldehyde
				Single coil BLU and V2					
1 *	1/M	BLU	Menthol	0.21 ± 0.05	0.21 ± 0.05	0.012 ± 0.003	0.03 ± 0.01	0.032 ± 0.002	BDL
2 *	2/F	BLU	Menthol	0.21 ± 0.05	0.21 ± 0.05	0.012 ± 0.003	0.03 ± 0.01	0.032 ± 0.002	BDL
3 *	3/F	BLU	Classic	10.6 ± 3.8	1.95 ± 0.35	0.22 ± 0.03	1.32 ± 0.37	0.072 ± 0.007	BDL
4 *	4/F	BLU	Classic	0.36 ± 0.04	0.33 ± 0.07	0.015 ± 0.003	0.038 ± 0.007	0.04 ± 0.01	BDL
5 *	5/M	BLU	Classic	0.36 ± 0.04	0.33 ± 0.07	0.015 ± 0.003	0.038 ± 0.007	0.04 ± 0.01	BDL
6	6/F	V2	Red Tobacco	1.55 ± 0.07	1.40 ± 0.13	0.034 ± 0.007	0.30 ± 0.24	0.31 ± 0.06	BDL
				Top single coil CE4					
7	7/M	CE4	Bubble gum	24.4 ± 2.3	22.5 ± 6.2	1.37 ± 0.35	0.85 ± 0.16	4.2 ± 1.2	BDL
8	8/M	CE4	Watermelon	0.49 ± 0.16	0.12 ± 0.10	BDL	0.18 ± 0.07	0.019 ± 0.008	0.16 ± 0.02
9	9/F	CE4	Watermelon	1.95 ± 0.39	0.39 ± 0.08	0.028 ± 0.006	0.84 ± 0.17	0.033 ± 0.002	0.19 ± 0.04
10	10/F	CE4	Watermelon	4.18 ± 1.34	1.21 ± 0.80	0.13 ± 0.09	1.62 ± 0.39	0.10 ± 0.07	0.11 ± 0.03
				Three-battery vaporizers: Aspire Cleito and Sigelei					
11 *	6/F	Aspire Cleito	Watermelon	0.41 ± 0.06	0.16 ± 0.03	0.058 ± 0.006	BDL	0.020 ± 0.004	0.36 ± 0.05
12 *	7/M	Sigelei	Fruit mix	4.59 ± 0.99	0.43 ± 0.09	BDL	0.69 ± 0.19	0.19 ± 0.04	BDL
13 *	7/M	Aspire Cleito	PG/VG	0.21 ± 0.03	0.025 ± 0.001	BDL	BDL	BDL	BDL
14 *	7/M	Aspire Cleito	PG/VG/nicotine	0.13 ± 0.03	0.022 ± 0.008	BDL	0.019 ± 0.004	BDL	BDL
15 *	7/M	Sigelei	Vanilla	0.31 ± 0.06	0.15 ± 0.03	0.09 ± 0.02	BDL	0.038 ± 0.008	BDL
16 *	11/F	Aspire Cleito	Butterspot	0.17 ± 0.03	0.21 ± 0.06	BDL	0.77 ± 0.23	12.1 ± 2.7	0.62 ± 0.09
17 *	12/M	Aspire Cleito	Snozberry	0.059 ± 0.006	0.19 ± 0.03	0.034 ± 0.007	BDL	0.18 ± 0.04	3.9 ± 1.2
18 *	7/M	Sigelei	Vanilla+fruit	0.23 ± 0.05	0.12 ± 0.02	BDL	BDL	0.047 ± 0.009	0.48 ± 0.09
19 *	7/M	Sigelei	Vanilla+fruit	-	-	-	-	-	-

*—participants used personal e-cigarette and e-liquid; concentrations of aldehydes (in µg·puff^−1^) measured in direct e-cigarette emissions; one male volunteer (participant #7) participated seven times and one female volunteer (participant #6) participated two times; BDL—below detection limit, PG—propylene glycol, VG—vegetable glycerol.

## Supplementary Materials

The following are available online at http://www.mdpi.com/2305-6304/6/3/46/s1, Table S1: Used e-cigarette devices, Table S2: Difference (Δ*C*) in carbonyl concentrations between exhaled e-cigarette breath (*C_e-cig breath_*) and background breath (*C_background_*), Table S3: Level of exposure to different aldehydes, Table S4: Exposure levels in mg·m^−3^ for 10 min, Table S5: Spearman correlations between elevated aldehyde levels in exhales e-cigarette breath (∆*C_aldehyde_*) for three groups of e-cigarettes, Figure S1: Sampling systems for collection of (a) exhaled breath and (b) mainstream e-cigarette emissions, Figure S2: Propionaldehyde concentrations in (a) “vape” breath (∆*C* = *C_e-cig breath_* − *C_background_*) and (b) direct e-cigarette emissions, Figure S3: Fraction of formaldehyde and acetaldehyde retained by human RT measured for one male volunteer, Figure S4: Correlations between elevated aldehyde levels in exhales e-cigarette breath (∆*C_aldehyde_*) and aldehyde concentration in mainstream of e-cigarette aerosol.
