# Supplementary material for: Aldehydes in Exhaled Breath during E-Cigarette Vaping: Pilot Study Results
Source: Toxics. 2018 Aug 7;6(3):46. doi: 10.3390/toxics6030046 (PMC6161081; doi:10.3390/toxics6030046)
Supplement: Supplementary file 1 [file toxics-06-00046-s001.docx]

**Supplementary Materials: Aldehydes in Exhaled Breath during E-Cigarette Vaping: Pilot Study Results**

Vera Samburova, Chiranjivi Bhattarai, Matthew Strickland, Lyndsey Darrow, Jeff Angermann, Yeongkwon Son and Andrey Khlystov

**Keywords:** Electronic cigarettes; aldehydes; breath analysis; respiratory tract retention; exposure

Experimental Section

The breakthrough was checked for several e-cigarettes using the second DNPH cartridge. Except for CE-4 e-cigarette, which generated the highest concentration of aldehydes, the concentration levels of aldehydes collected on the second “breakthrough” DNPH cartridge were similar to the background levels. In case of CE-4, for some flavoring e-liquids (e.g., Bubble Gum) carbonyls’ breakthrough was up to 20%.

**Table S1.** Used e-cigarette devices.

| **Session #** | **E-cigarette *** | **Flavor *** | **Battery Brand** | **Type** | **PG/VG (*v/v*)** | **Nicotine (mg·mL^−1^)** | **Power (W)** |
| --- | --- | --- | --- | --- | --- | --- | --- |
| 1 | BLU | Menthol | Built-in | Cigarette like device | 0/100 | 18 | 4.75 |
| 2 | BLU | Menthol | Built-in | Single coil | 0/100 | 18 | 4.75 |
| 3 | BLU | Classic | Built-in | - | 0/100 | 18 | 4.75 |
| 4 | BLU | Classic | Built-in | - | 0/100 | 18 | 4.75 |
| 5 | BLU | Classic | Built-in | - | 0/100 | 18 | 4.75 |
| 6 | V2 | Red Tobacco | Built-in | - | 80/20 | 18 | 5.2 |
| 7 | CE4 | Bubble gum | eGo | 2^nd^ generation | 80/20 | 12 | 4.9 |
| 8 | CE4 | Watermelon | eGo | Top single coil | 80/20 | - | - |
| 9 | CE4 | Watermelon | eGo | - | 80/20 | - | - |
| 10 | CE4 | Watermelon | eGo | - | 80/20 | - | - |
| 11 | Aspire Cleito | Watermelon | Reuleaux RX200 | 3^rd^ generation | 40/60 | 6 | 50 |
| 12 | Sigelei | Fruit mix | Sigelei | Three-battery vaporizer | N/A | 6–20 | 80–100 |
| 13 | Aspire Cleito | PG/VG | Reuleaux RX200 | - | N/A | 6 | 90 |
| 14 | Aspire Cleito | PG/VG/nicotine | Reuleaux RX200 | - | N/A | 6 | 90 |
| 15 | Segelei | Vanilla | Sigelei | - | 70/30 | 6–20 | 80–100 |
| 16 | Aspire Cleito | Butterspot | Reuleaux RX200 | - | N/A | 6–20 | 80–100 |
| 17 | Aspire Cleito | Snozberry | Reuleaux RX200 | - | N/A | 6–20 | 80–100 |
| 18 | Sigelei | Vanilla+fruit | Sigelei | - | N/A | 6–20 | 80–100 |
| 19 | Sigelei | Vanilla+fruit | Sigelei | - | N/A | 6–20 | 80–100 |

*—e-liquids and/built-in e-cigarettes (BLU and V2) were purchased several days prior to use; e-liquids and cartridges with e-liquids were kept at room temperature in closed cabinets; only non-expired e-liquids and premade.

**Table S2.** Difference (Δ*C*) in carbonyl concentrations between exhaled e-cigarette breath (*C_e-cig breath_*) and background breath (*C_background_*).

| **Sessions #** | **Formaldehyde** | **Acetaldehyde** | **Acrolein** | **Glyoxal** | **Propionaldehyde** | **MEK** | **Valeraldehyde** | **Benzaldehyde** |
| --- | --- | --- | --- | --- | --- | --- | --- | --- |
| 1 | −0.001 | 0.028 | 0.000 | 0.000 | 0.014 | 0.000 | 0.000 | 0.000 |
| 2 | −0.001 | 0.023 | 0.000 | 0.000 | 0.000 | 0.000 | 0.000 | 0.000 |
| 3 | 0.001 | −0.009 | 0.000 | 0.000 | 0.000 | 0.000 | 0.000 | 0.000 |
| 4 | 0.000 | 0.031 | 0.000 | 0.000 | 0.099 | 0.000 | 0.000 | 0.000 |
| 5 | 0.000 | 0.003 | 0.000 | 0.000 | 0.000 | 0.000 | 0.000 | 0.000 |
| 6 | 0.019 | 0.055 | 0.000 | 0.000 | 0.010 | 0.000 | 0.000 | 0.000 |
| 7 | 0.037 | 0.021 | 0.000 | 0.000 | 0.000 | 0.000 | 0.000 | 0.000 |
| 8 | 0.408 | 0.555 | 0.000 | 0.072 | 0.129 | 0.101 | 0.000 | 0.000 |
| 9 | 0.012 | 0.017 | 0.000 | 0.000 | 0.044 | 0.000 | 0.000 | 0.000 |
| 10 | 0.072 | 0.021 | 0.000 | 0.000 | 0.045 | 0.000 | 0.000 | 0.000 |
| 11 | 0.003 | 0.004 | 0.000 | 0.000 | 0.043 | 0.000 | 0.000 | 0.013 |
| 12 | 0.083 | 0.104 | 0.000 | 0.036 | 0.159 | 0.000 | 0.000 | 0.000 |
| 13 | −0.002 | 0.000 | 0.000 | 0.000 | 0.034 | 0.000 | 0.000 | 0.000 |
| 14 | −0.002 | −0.011 | 0.000 | 0.000 | 0.019 | 0.000 | 0.000 | 0.000 |
| 15 | 0.006 | 0.041 | 0.000 | 0.000 | 0.063 | 0.000 | 0.000 | 0.000 |
| 16 | 0.000 | 0.005 | 0.000 | 0.000 | 1.054 | 0.000 | 0.000 | 0.079 |
| 17 | 0.000 | 0.006 | 0.000 | 0.000 | 0.352 | 0.000 | 0.000 | 0.181 |
| 18 | −0.001 | 0.030 | 0.000 | 0.000 | 0.026 | 0.000 | 0.000 | 0.000 |
| 19 | 0.001 | −0.007 | 0.000 | 0.000 | 0.041 | 0.000 | 0.000 | 0.007 |

units: µg·breath^−1^.

**Table S3.** Level of exposure to different aldehydes.

| **Session #** | **Formaldehyde  μg·puff^−1^** | **Acetaldehyde  μg·puff^−1^** | **Acrolein  μg·puff^−1^** | | **Glyoxal  μg·puff^−1^** | **Propionaldehyde  μg·puff^−1^** | **Benzaldehyde  μg·puff^−1^** | **Total Carbonyl Exposure  μg·puff^−1^** |
| --- | --- | --- | --- | --- | --- | --- | --- | --- |
| 1 | 0.212 | 0.142 | 0.012 | 0.030 | | 0.018 | 0.000 | 0.414 |
| 2 | 0.213 | 0.147 | 0.012 | 0.030 | | 0.032 | 0.000 | 0.433 |
| 3 | 10.605 | 1.958 | 0.224 | 1.324 | | 0.072 | 0.000 | 14.183 |
| 4 | 0.363 | 0.303 | 0.015 | 0.038 | | −0.062 | 0.000 | 0.657 |
| 5 | 0.363 | 0.331 | 0.015 | 0.038 | | 0.038 | 0.000 | 0.785 |
| 6 | 1.530 | 1.346 | 0.034 | 0.299 | | 0.301 | 0.000 | 3.510 |
| 7 | 24.401 | 22.445 | 1.373 | 0.848 | | 4.175 | 0.000 | 53.242 |
| 8 | 0.078 | −0.433 | 0.000 | 0.107 | | −0.109 | 0.161 | −0.196 |
| 9 | 1.941 | 0.372 | 0.028 | 0.840 | | −0.011 | 0.192 | 3.363 |
| 10 | 4.110 | 1.191 | 0.128 | 1.622 | | 0.055 | 0.108 | 7.215 |
| 11 | 0.411 | 0.154 | 0.058 | 0.000 | | −0.023 | 0.343 | 0.944 |
| 12 | 4.509 | 0.329 | 0.000 | 0.658 | | 0.029 | 0.000 | 5.525 |
| 13 | 0.207 | 0.026 | 0.000 | 0.000 | | −0.034 | 0.000 | 0.198 |
| 14 | 0.132 | 0.033 | 0.000 | 0.019 | | −0.019 | 0.000 | 0.165 |
| 15 | 0.303 | 0.107 | 0.086 | 0.000 | | −0.025 | 0.000 | 0.471 |
| 16 | 0.172 | 0.202 | 0.000 | 0.771 | | 11.061 | 0.539 | 12.745 |
| 17 | 0.059 | 0.185 | 0.034 | 0.000 | | −0.172 | 3.739 | 3.845 |
| 18 | 0.230 | 0.087 | 0.000 | 0.000 | | 0.020 | 0.483 | 0.820 |
| 19 | NA | NA | NA | NA | | NA | NA | NA |

**Table S4.** Exposure levels in mg·m^−3^ for 10 min.

| **Session #** | **Formaldehyde** | **Acetaldehyde** | **Acrolein** | **Glyoxal** | **Propionaldehyde** | **Benzaldehyde** |
| --- | --- | --- | --- | --- | --- | --- |
| 1 | 0.039 | 0.031 | 0.002 | 0.005 | 0.006 | 0.000 |
| 2 | 0.039 | 0.031 | 0.002 | 0.005 | 0.006 | 0.000 |
| 3 | 1.928 | 0.354 | 0.041 | 0.241 | 0.013 | 0.000 |
| 4 | 0.066 | 0.061 | 0.003 | 0.007 | 0.007 | 0.000 |
| 5 | 0.066 | 0.061 | 0.003 | 0.007 | 0.007 | 0.000 |
| 6 | 0.282 | 0.255 | 0.006 | 0.054 | 0.056 | 0.000 |
| 7 | 4.443 | 4.085 | 0.250 | 0.154 | 0.759 | 0.000 |
| 8 | 0.089 | 0.022 | 0.000 | 0.033 | 0.004 | 0.029 |
| 9 | 0.355 | 0.071 | 0.005 | 0.153 | 0.006 | 0.035 |
| 10 | 0.760 | 0.220 | 0.023 | 0.295 | 0.018 | 0.020 |
| 11 | 0.075 | 0.029 | 0.011 | 0.000 | 0.004 | 0.065 |
| 12 | 0.835 | 0.079 | 0.000 | 0.126 | 0.034 | 0.000 |
| 13 | 0.037 | 0.005 | 0.000 | 0.000 | 0.000 | 0.000 |
| 14 | 0.024 | 0.004 | 0.000 | 0.003 | 0.000 | 0.000 |
| 15 | 0.056 | 0.027 | 0.016 | 0.000 | 0.007 | 0.000 |
| 16 | 0.031 | 0.038 | 0.000 | 0.140 | 2.203 | 0.112 |
| 17 | 0.011 | 0.035 | 0.006 | 0.000 | 0.033 | 0.713 |
| 18 | 0.042 | 0.021 | 0.000 | 0.000 | 0.008 | 0.088 |
| 19 | 0.000 | 0.000 | 0.000 | 0.000 | 0.000 | 0.000 |

To calculate the exposure the aldehyde levels were multiplied by 20 puffs (averaged number of puffs per e-cigarette use session) and divided by 0.11 m^3^—inhaled volume of air per 10 min for an adult (16 m^3^—inhaled volume of air per 24 h for an adult). The obtained levels were compared with available Acute Exposure Guideline Levels (AEGL-1) [1]: formaldehyde—1.1 mg·m^−3^ for 10 min exposure, acetaldehyde—81 mg·m^−3^, and acrolein—0.07 mg·m^−3^.

**Table S5.** Spearman correlations between elevated aldehyde levels in exhales e-cigarette breath (∆*C_aldehyde_*) for three groups of e-cigarettes.

| E-cigarette | **Formaldehyde** | **Acetaldehyde** | **Propionaldehyde** |
| --- | --- | --- | --- |
| BLU and V2 | *r* = 0.948 (*p* = 0.013) | *r* = 0.527 (*p* = 0.362) | *r* = −0.063 (*p* = 0.906) |
| CE4 | *r* = −0.400 (*p* = 0.750) | *r* = −0.200 (*p* = 0.917) | *r* = −0.800 (*p* = 0.330) |
| Aspire Cleito and Segelei | *r* = 0.695 (*p* = 0.056) | *r* = −0.619 (*p* = 0.115) | *r* = 0.495 (*p* = 0.213) |

**Figure S1.** Sampling systems for collection of **a**) exhaled breath and **b**) mainstream e-cigarette emissions.

**
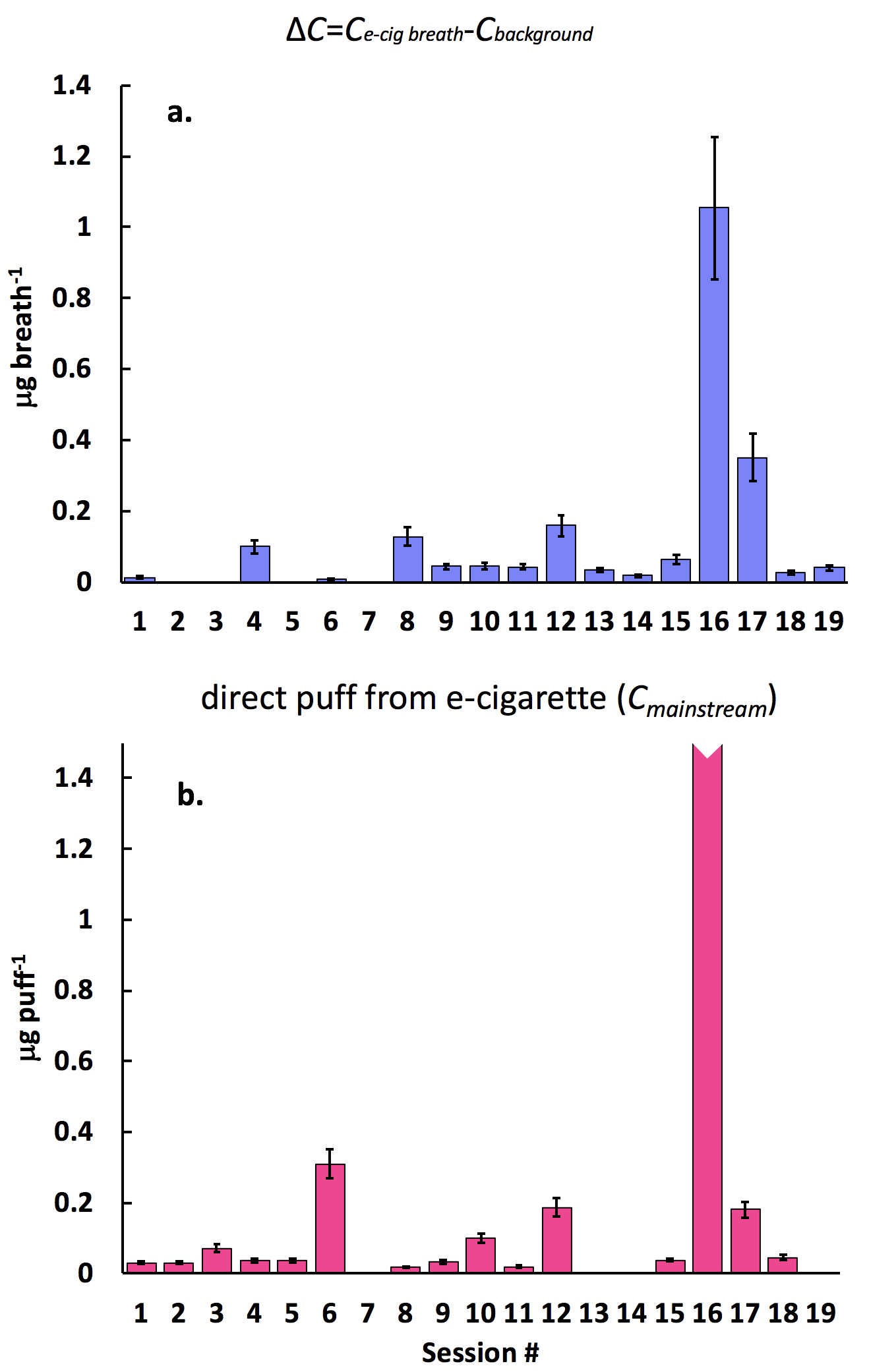
**

**Figure S2.** Propionaldehyde concentrations in (**a**) “vape” breath (∆*C* = *C_e-cig breath_ −* *C_background_*) and (**b**) direct e-cigarette emissions; the mean values of standard deviations for propionaldehyde in direct e-cigarette emissions and ∆*C* were 23 ± 14% and 19 ± 14%, respectively.

**Figure S3.** Fraction of formaldehyde and acetaldehyde retained by human RT measured for one male volunteer (number of replicates: *n* = 9).

**Figure S4.** Correlations between elevated aldehyde levels in exhales e-cigarette breath (∆*C_aldehyde_*) and aldehyde concentration in mainstream of e-cigarette aerosol.

References

1. National Research Council*.* Committee on acute exposure guideline levels. In *Acute Exposure Guideline Levels for Selected Airborne Chemicals*; National Academies Press: Washington, DC, USA, 2008; Volume 8.
